# Regional registration of whole slide image stacks containing major histological artifacts

**DOI:** 10.1186/s12859-020-03907-6

**Published:** 2020-12-04

**Authors:** Mahsa Paknezhad, Sheng Yang Michael Loh, Yukti Choudhury, Valerie Koh Cui Koh, Timothy Tay Kwang Yong, Hui Shan Tan, Ravindran Kanesvaran, Puay Hoon Tan, John Yuen Shyi Peng, Weimiao Yu, Yongcheng Benjamin Tan, Yong Zhen Loy, Min-Han Tan, Hwee Kuan Lee

**Affiliations:** 1 Imaging Informatics Division, Bioinformatics Institute (BII), Agency for Science, Technology and Research (A*STAR), 30 Biopolis Street, 07-01, Matrix, 138671 Singapore, Singapore; 2 National University of Singapore, 21 Lower Kent Ridge Rd, 119077 Singapore, Singapore; 3 Lucence Diagnostics, 217 Henderson Road, 03-08, Henderson Industrial Park, 159555 Singapore, Singapore; 4 Institute of Bioengineering and Nanotechnology, 31 Biopolis Way, The Nanos 09-01, 138669 Singapore, Singapore; 5 Singapore General Hospital, Outram Road, 169608 Singapore, Singapore; 6 National Cancer Centre Singapore, 11 Hospital Drive, 169610 Singapore, Singapore; 7 Institute of Molecular and Cell Biology, 61 Biopolis Drive, 138673 Singapore, Singapore; 8 Image and Pervasive Access Lab (IPAL), CNRS UMI 2955, 1 Fusionopolis Way, 138632 Singapore, Singapore; 9 Singapore Eye Research Institute, 20 College Road, 169856 Singapore, Singapore

**Keywords:** Whole slide images, Immunohistochemistry images, Rigid registration, Blood vessel 3D reconstruction, Multi-scale attention

## Abstract

**Background:**

High resolution 2D whole slide imaging provides rich information about the tissue structure. This information can be a lot richer if these 2D images can be stacked into a 3D tissue volume. A 3D analysis, however, requires accurate reconstruction of the tissue volume from the 2D image stack. This task is not trivial due to the distortions such as tissue tearing, folding and missing at each slide. Performing registration for the whole tissue slices may be adversely affected by distorted tissue regions. Consequently, regional registration is found to be more effective. In this paper, we propose a new approach to an accurate and robust registration of regions of interest for whole slide images. We introduce the idea of multi-scale attention for registration.

**Results:**

Using mean similarity index as the metric, the proposed algorithm (mean ± SD $0.84 \pm 0.11$) followed by a fine registration algorithm ($0.86 \pm 0.08$) outperformed the state-of-the-art linear whole tissue registration algorithm ($0.74 \pm 0.19$) and the regional version of this algorithm ($0.81 \pm 0.15$). The proposed algorithm also outperforms the state-of-the-art nonlinear registration algorithm (original: $0.82 \pm 0.12$, regional: $0.77 \pm 0.22$) for whole slide images and a recently proposed patch-based registration algorithm (patch size 256: $0.79 \pm 0.16$ , patch size 512: $0.77 \pm 0.16$) for medical images.

**Conclusion:**

Using multi-scale attention mechanism leads to a more robust and accurate solution to the problem of regional registration of whole slide images corrupted in some parts by major histological artifacts in the imaged tissue.

## Background

This section presents the motivation for proposing a regional registration algorithm for whole slides images (WSIs). It also provides the objectives in this paper, literature review of the previously proposed registration algorithms for WSIs, and the innovation in the proposed algorithm compared to existing registration methods.

### Motivation

Whole slide imaging (WSI) has enabled developing automatic analysis and diagnostic tools for more accurate identification and study of diseases. Since its development, clinical studies using WSIs and light microscopy have shown comparable accuracy for primary diagnostics in breast pathology [1], gastrointestinal tract pathology [2], and urinary system pathology [3]. High-resolution 3D reconstruction of the original tissue volume from the 2D slices of WSIs enables researchers to study certain features which cannot be revealed inthe 2D images, such as the vascular structure, length and branching of vessels or colocalization of biomarkers. During the tissue cutting and mounting process, each individual thin section may experience serious artifacts such as tissue tearing, folding, or missing. As a result, accurate 3D reconstruction of the tissue volume requires first registering the tissue in subsequent image slides.

**Fig. 1:**
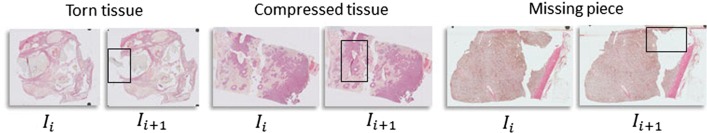
A few examples of tissue deformation in two consecutive whole slide images ($I_i$, $I_{i+1}$). Such deformations make registration of whole slide images challenging

The image acquisition process for a small volume of fixed tissue involves cutting the volume into very thin sections and mounting them on microscope slides for staining. Scanning is performed for each slide individually after staining. The morphological changes which may occur to the tissue during slide preparation such as tissue compression or stretching, missing or torn tissue, stain variations, rotation and translation of the tissue [4] are some of the reasons why registration of whole slide images is challenging. Figure 1 shows a few examples of tumor tissue samples from patients with clear cell renal cell carcinoma. Tissue compression, missing tissue and tissue tears are found very often in the scanned images. Currently, there exists no algorithm which can recover the highly deformed tissue regions shown with black boxes in Fig. 1. This is also not the aim of the proposed registration algorithm in this paper. These deformed regions may occupy a large part of the image and adversely affect many global registration algorithms [5–13] making these algorithms unreliable. The aim of this paper is to address this problem by designing a robust registration algorithm that registers user-defined tissue regions in WSIs accurately. This registration method is not affected by the presence of adverse artifacts outside of the region of interest (ROI).

### Objectives

We summarize the objectives of this paper as follows:

1. Design a registration algorithm that can perform registration for the ROI defined by the user in the WSI stack with high accuracy.
2. Registration accuracy of the proposed algorithm will not be affected by any highly deformed regions outside of the selected ROI.
3. The proposed algorithm can perform registration in a reasonable amount of time for hundreds of WSIs.
4. The proposed algorithm is highly robust in the existence of different types of distortions in the WSI stack.

### Literature review

Due to the large size of whole slide images in their full resolution, well-established registration methods cannot be deployed to register these images with high accuracy without a high performance computing system. Application of these methods on lower resolutions may also result in significant registration errors in the full resolution. Previously proposed whole slide tissue registration algorithms take different approaches to keep the computation time reasonable as well as to find the global optimum solution. Proposed algorithms such as [5, 6, 8] are classified as multi-scale approaches. In multi-scale registration methods, the registration is performed for coarse resolutions of the images first and the resulting deformation field is refined using finer resolutions of the images. In the work by Wang and Chen [6], for instance, the images are sparsely represented in the coarse level by extracting SIFT features [14]. The extracted features are used to estimate the transformation needed to align the two images. In the fine level, an area based b-spline method is deployed to improve the registration. In another work by Moles Lopez et al. [5], a four-level pyramidal registration with linear transformations is utilized. To increase the registration speed, similarity is only measured on randomly sampled pixels from the whole tissue in this approach.

The works such as [10–12] are patch-based approaches to WSI registration. In patch-based methods, the image is divided into regularly spaced patches and registration is carried out for each patch individually. The method proposed by Balakrishnan et al. [7, 15] is a good example of this group of algorithms. In this algorithm, a convolutional neural network is trained on randomly selected pairs of moving and fixed image patches from the training data set in an unsupervised manner using a loss function that takes into account image similarity and deformation field smoothness. The trained network is then able to register unseen whole tissue image pairs. Many patch-based algorithms are also multi-scale. The work by Roberts et al. [10], is a good example, where consecutive slides are aligned non-rigidly first. Patch-based registration is then performed for increasing resolutions of the images. Finally, a global b-spline non-rigid transformation is estimated from the set of rigid patch transforms. The work by Lotz et al. [12] is another example of multi-scale patch-based methods where non-rigid registration is carried out on the patches that are allowed to overlap. Proposed registration algorithms such as [9, 13] take advantage of the vessel structure in images to improve the registration output. In the work by Schwier at al. [13], for example, vessels are extracted from each image and non-rigid registration is performed using the vessel masks. The work by Liang et al. [9] proposes a multi-scale registration algorithm which rigidly aligns the patches, fuses the rigid transformations by a cubic b-spline deformation and performs vessel segmentation and association later on to reduce the registration error. The work by Jiang et al. [16] presents a registration algorithm for registering re-stained images which refers to the procedure when tissue is stained, imaged, washed, stained again, and imaged again. In this procedure, the tissue slices do not suffer from potential distortions related to sectioning, or differences in the tissue sections. Consequently, they fulfill a different objective from our objective in this paper.

Considering the strong presence of tissue deformations in the acquired virtual slices and the large size of these images, landmark-based methods may be misguided by highly deformed regions in the tissue. The work by Vink et al. [17] suggests that in order to get an accurate registration of whole slide images, landmarks located in unreliable areas, such as folded, torn or missing tissue regions, need to be detected and treated differently from reliable landmarks. There has been efforts to detect unreliable regions in the tissue such as the work by Babaie et al. [18] where folded tissue regions are detected using a deep learning technique or the work by Agarwal et al. [19] where an algorithm is proposed to locate major histological artifacts in tissue images. However, these algorithms are trained or tested on specific image data sets. Performance of landmark-based registration algorithms using these artifact detection methods need to be evaluated. Solorzano et al. [20] divide the whole tissue slide into manually selected sub-regions and perform registration in a single scale for each sub-region using coarse features. Although performing registration on tissue sub-regions improves the registration results, single-scale registration does not yield high cell-level registration accuracy. Also, registration for regions with highly deformed tissue sections will be poor and may adversely affect the output deformation field for the whole tissue slide. We propose a novel multi-scale approach for registration of whole slide images that performs registration only for the user-defined ROI on the tissue section. The proposed algorithm registers the ROI marked by the user in the tissue section very well. The novelty is the multi-scale attention mechanism which makes the registration algorithm robust and accurate. Artifacts outside of the ROI marked by the user do not affect the registration outcome. A naive solution to registration of the user-defined ROI is to crop the ROI in the 2D image stack and perform registration for the cropped regions. The cropped regions, however, may not have enough common tissue information based on which an accurate registration could be achieved. We present our proposed algorithm in Method section. We have focused on registration of the region around blood vessels in order to be able to quantify the registration accuracy using manual lumen segmentations. In the next section, we evaluate the proposed method on a data set of whole slide image stacks acquired from clear cell renal cell carcinoma patients.

### Innovation

Although there are proposed algorithms that take advantage of (1) multi-scale registration techniques [5] and (2) coarse features in the tissue slides [6] for registration of WSIs, the novelty of the proposed algorithm in this paper lies on the efficient way these two factors are deployed. The first novelty compared to the previously proposed multi-scale registration algorithms is in the fact that we use a multi-scale attention mechanism where registration is initiated for a large region around the ROI and increasingly concentrates on a smaller region around the ROI as the resolution increases, giving more attention to a smaller region around the ROI. This is in contrast to the existing multi-scale algorithms where the whole tissue is registered at all resolutions. Registration starts on the entire low resolution images and proceeds to registering the entire high resolution images. Our mutli-scale attention approach makes the registration robust against major deformations that may exist outside of the ROI. It also makes the algorithm desirable for registering a ROI in large images in a reasonable amount of time. The second novelty is that we propose a method to select a small subset of SIFT key points that can effectively align the two tissue regions. Therefore, our registration algorithm avoids using non-specific key points that would degrade the registration outcome. We will explain our algorithm in detail in the Method Section. These two approaches together deliver a robust regional registration technique which satisfies the objectives of this paper as mentioned in the Objectives Section. The proposed algorithm does not need to know the location of highly deformed regions in the tissue section.

## Results

In this section, we evaluate the performance of the proposed registration algorithm on our data set of whole immunohistochemical slice images of clear cell renal cell carcinoma. The organization of this section is as follows. We explain our data set in the following paragraph, followed by the evaluation of the proposed algorithm on the presented data set. Next, we compare our proposed algorithm with three different previously proposed methods: (1) a multi-scale rigid registration algorithm [5], (2) a patch-based, deep learning registration algorithm [7], and (3) a SIFT-feature-based non-rigid registration algorithm [6]. The mentioned algorithms were found after an excessive search for a diverse set of well-known algorithms that address similar objectives in our paper.

### Data set

Our data set consists of whole immunohistochemical slide images of three patients with clear cell renal cell carcinoma. All three specimens were fixed in formalin and embedded in paraffin blocks. A sequence of 100 slices were cut with image resolution of 0.5 $\upmu \hbox {m}$/pixel for patient 1, and 0.25 $\upmu \hbox {m}$/pixel for patient 2, and 150 slices were cut with image resolution of 0.25 $\upmu \hbox {m}$/pixel for patient 3 from the tissue blocks with 4 $\upmu \hbox {m}$ thickness. All tissue sections were double-stained to reveal the endothelial cell marker CD31 (Dako M0823, Clone JC70A, 1:50 dilution, Epitope Retrieval 2, pH 9.0) and the pericyte marker $\alpha$-SMA Dako M0851, Clone 1A4, 1:1000 dilution, Epitope Retrieval 2, pH 9.0). Immunohistochemical staining was performed using the Leica Bond Max autostainer (Leica Biosystems Melbourne) according to the manufacturer’s instructions. The double stains were visualized using the Bond Polymer Refine Detection and the Bond Refine Red Detection systems (Leica Biosystems), with CD31 staining brown and SMA staining red. Each slide was scanned at high resolution with IntelliSite Pathology Ultra Fast Scanner (Philips Digital Pathology Solutions, The Netherlands) and viewed with IMS Viewer (Philips, The Netherlands). All registration experiments were performed on level 4 magnification of the whole slide images as the full resolution image in this paper. Although our data set consists of merely three acquired image stacks, a combination of 20 blood vessels with different shape and orientation and from different regions in the images were selected for registration to adequately test robustness of the proposed algorithm. Tissue tearing, folding, and missing were observed frequently in all 4 image stacks. Four resolution levels were considered for the proposed registration algorithm. The lumen for each blood vessel was segmented manually. The output transformation matrices for the registered blood vessels were applied to the corresponding lumen segmentations to measure the registration accuracy. It is important to emphasize that the lumen segmentations were solely used to compare the performance of the proposed method with the competing methods. These segmentations were not used in any steps of the registration pipeline.

### Evaluation of the proposed algorithm

**Fig. 2:**
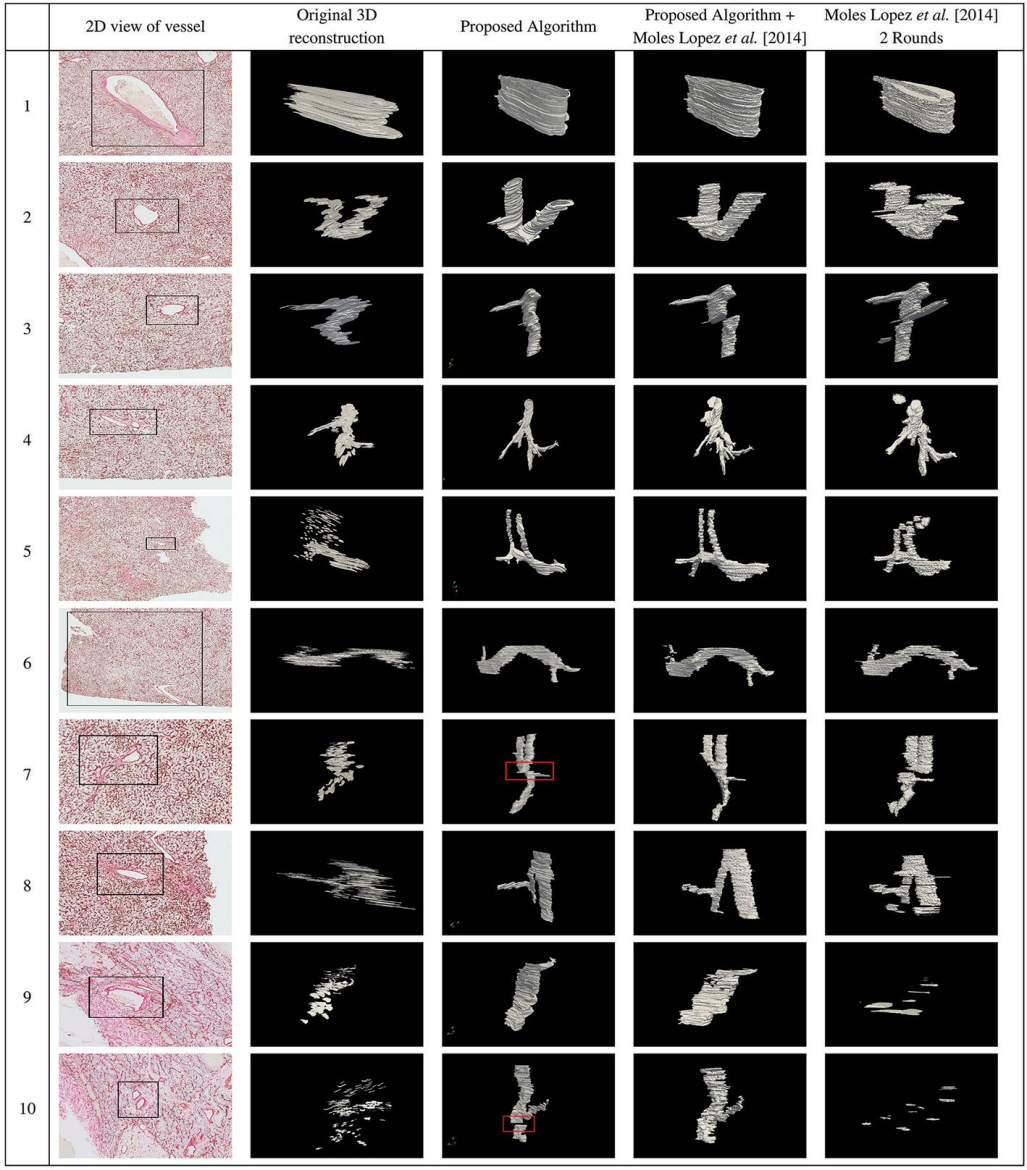
First column in figure shows a 2D view of the blood vessels that were chosen for registration. The second column shows 3D reconstruction of the blood vessels before registration. The 3D reconstruction of the blood vessels after registration using the proposed algorithm, the proposed algorithm followed by Moles Lopez et al. [5], and Moles Lopez et al. [5] 2 Rounds are shown in the third, fourth, and fifth columns, respectively

**Fig. 3:**
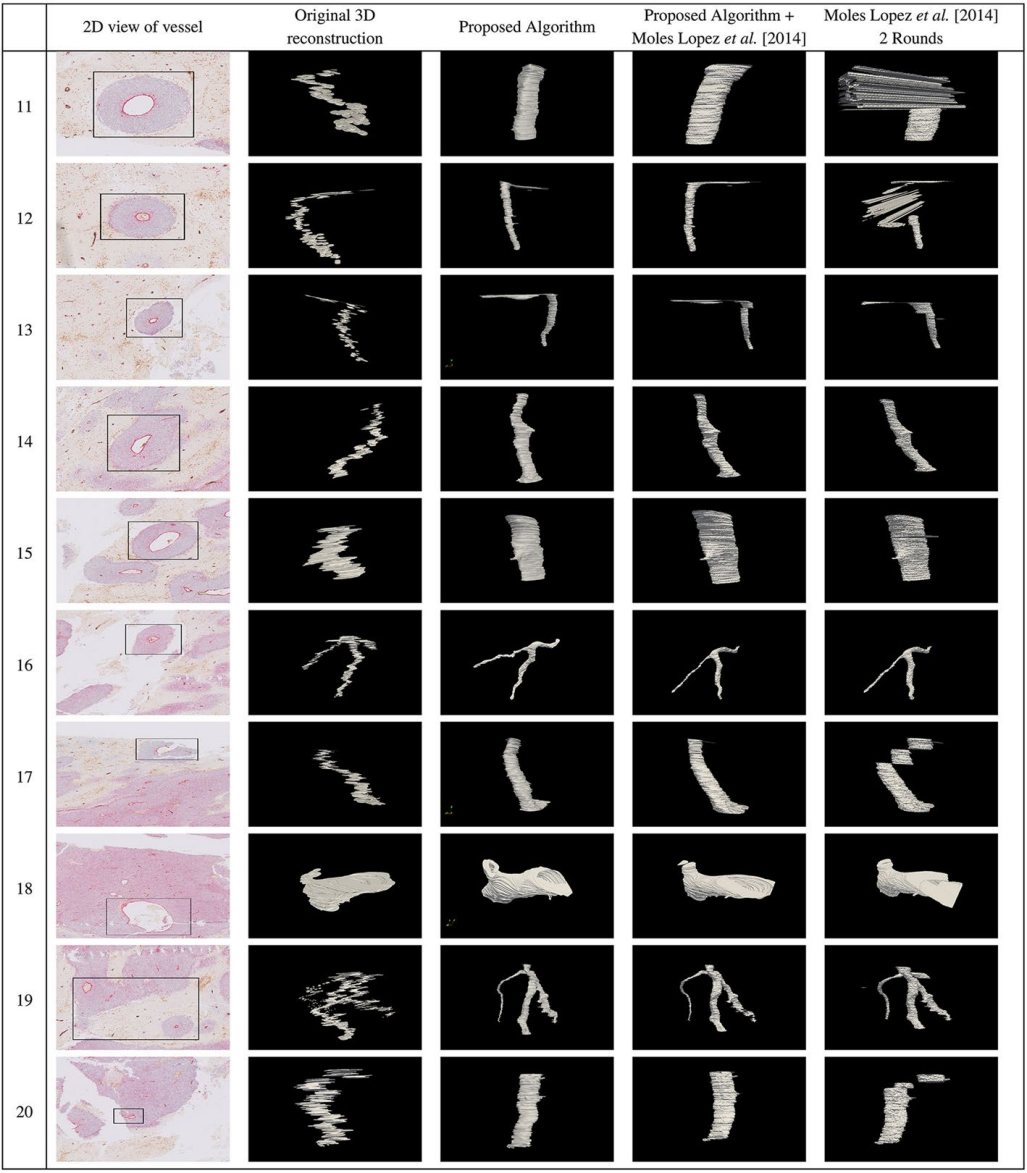
First column in figure shows a 2D view of the blood vessels that were chosen for registration. The second column shows 3D reconstruction of the blood vessels before registration. The 3D reconstruction of the blood vessels after registration using the proposed algorithm, the proposed algorithm followed by Moles Lopez et al. [5], and Moles Lopez et al. [5]. 2 Rounds are shown in the third, fourth, and fifth columns, respectively

A 2D view of the blood vessels that were selected for registration are shown in the first column in Figs. 2 and 3 for 20 blood vessels. We applied the proposed algorithm on all 20 selected blood vessels. The 3D reconstruction of the selected blood vessels before registration, and the resulting 3D reconstructed blood vessels after performing registration using the proposed algorithm are shown in the second and third columns, respectively, in Figs. 2 and 3. The original image scale in the z dimension is compressed for presentation purposes. As can be seen, the vessels are well-aligned using the proposed algorithm for most of the slices except for a few slices highlighted by red boxes. In Additional file 1: Fig. 3, we have provided the registration results for a few pairs of slides for these blood vessels, the best SIFT feature matches for the ROIs in the previous and current slides, and the selected 3 SIFT feature matches that resulted in the best alignment of the 2 slides.

In order to have a quantitative measurement of the accuracy of the proposed registration algorithm, we used Dice similarity coefficient as the similarity metric. Dice similarity coefficient (DSC) also known as Dice similarity index measures the similarity between non-zero pixels in the two lumen masks after registration using the following formula:

$$\begin{aligned} DSC = 2\frac{|A \bigcap B|}{|A| + |B|} \end{aligned}$$

where A and B are the set of non-zero pixels in the first and the second lumen mask, respectively. The operator |.| defines the size of the set and the operator $\bigcap$ represents the intersection of the two sets. This metric was chosen for quantitative analysis mainly because DSC stays meaningful for any two consecutive images in which blood vessel branching occurs. The DSC ($mean\pm std$) for lumen segmentations after registration using the proposed algorithm for the 20 blood vessels and 5 consecutive slices was measured and is reported as *Proposed Algorithm* in Table 2.

### Comparison with a multi-scale rigid approach

We compared our algorithm with the state-of-the-art multi-scale registration method by Moles Lopez et al. [5] mentioned in the Background Section where they introduce a four-level registration algorithm. Similar to our approach, their algorithm performs registration on the low resolution images first. The resulting deformation field is applied to the next higher resolution images before performing registration on this level. Their algorithm does not utilize the multi-scale attention mechanism proposed in this paper. In order to make the algorithm faster and more robust, they measure their similarity metric only on randomly sampled pixels in the whole image. They consider only linear transformations ,$T_\mu$, (affine and rigid) for registration. There exists a few hyperparameters for this algorithm such as the similarity metric: Mattes mutual information (MI) or normalized cross correlation (NCC), the number of pixels to evaluate the similarity metric on ($N_s$), the number of iterations, $N_i$, of the optimization procedure, and the image channels to use for registration: the hematoxylin channel (blue) or the luminance channel.

Table 1 shows the set of hyperparameters for this algorithm together with possible values for these parameters. The parameter values were taken from the set of values evaluated for high-resolution images in the paper by Moles Lopez et al. [5] (Table 1 in the paper) except for the values for maximum step length (MSL) where we found the range 1, 5, 10, 20 outputting more accurate registration results. We tested the algorithm with different combination of the parameter values defined in Table 1 for 5 consecutive slices and found the set of values that gave the best mean registration accuracy for all the 20 blood vessels to be $T_\mu =$ Affine, S = MI, MSL = 10, and Input = Lum.

**Table 1:** Table shows the set of parameter values that were tested for the multi-scale registration algorithm proposed by Moles Lopez et al. [5]

Parameter	All resolutions
$T_\mu$	*Affine*, Rigid
S	*MI*, NCC
$N_s$	*8000*
$N_i$	*2000*
MSL	1, 5, *10*, 20
Input	*Lum*, Hem

The parameter values which resulted in the best mean registration accuracy for all the 20 blood vessels are shown in italic

**Table 2:** Mean Dice similarity coefficient (DSC) for the lumen segmentations after registration

Method	DSC	Training time	Exec. time
Moles Lopez et al. [5]—1 Round	$0.74 \pm 0.19$	−	5.8 min^a^
Moles Lopez et al. [5]—2 Rounds	$0.81 \pm 0.15$	−	6.4min^a^
Wang and Chen [6]—1 Round	$0.82 \pm 0.12$	−	2.8 min ^b^
Wang and Chen [6]—2 Rounds	$0.77 \pm 0.22$	−	3.0 min^b^
Balakrishnan et al. [7]—Patch size 256	$0.79 \pm 0.16$	80.5 min^a^	0.34 min^a^
Balakrishnan et al. [7]—Patch size 512	$0.77 \pm 0.16$	316.9 min^a^	0.35 min^a^
Proposed Algorithm	$0.84 \pm 0.11$	−	3.4 min^a^
Proposed Algorithm followed by Moles Lopez et al. [5]	$\mathbf {0.86 \pm 0.08}$	−	5.6 min^a^

The DSC was measured for lumen segmentations of 20 blood vessels for 5 consecutive slices after registration using the method proposed by Moles Lopez et al. [5] (Moles Lopez et al. [5] 1 Round), the regional version of this method (Moles Lopez et al. [5] 2 Rounds), the method proposed by Wang and Chen [6] (Wang and Chen [6]—1 Round), the regional version of this method (Wang and Chen [6]—2 Rounds), and the patch-based method proposed by Balakrishnan et al. [7] with patch sizes 256 $\times$ 256 pixels and 512 $\times$ 512 pixels. Their performance was compared with those of the proposed algorithm, and the proposed method followed by fine registration using Moles Lopez et al. [5]. The time required for training and executing the algorithms on 5 consecutive image slices is presented in the third and fourth columns

^a^Ubuntu 19.04.4 LTS 64-bit, Intel Core i7-6700 CPU 3.40 GHz × 8, 31.4GB RAM

^b^Windows 8.1 Pro 64-bit, Intel Core i7-4720HQ CPU 2.60GHz, 11.9GB RAM

Using this set of values, we applied the method on the images with the surrounding artifacts removed ($\tilde{I}_{seq}$) for all the 20 blood vessels following the *Removing surrounding artifacts* steps in the Methods section. The mean registration accuracy for this method is reported as Moles Lopez et al. [5] 1 Round in Table. 2. To have a fair comparison, the method proposed by Moles Lopez et al. [5] was also provided with the same manual user input ($ROI_i^0$) that was provided to our algorithm. The ROI defined by user ($ROI_i^0$) was extracted from the registered images and further registration was performed on the cropped regions. This approach is referred to as (Moles Lopez et al. [5] 2 Rounds) in Table 2. After applying the proposed registration algorithm, the user-defined ROI was extracted from the registered images and another round of registration using the method by Moles Lopez et al. [5] was performed to further improve the registration accuracy. We refer to this method as *proposed method followed by fine registration* throughout the paper. Registration accuracy for the proposed algorithm followed by fine registration using the work of Moles Lopez et al. [5] is also reported in Table 2.

**Fig. 4:**
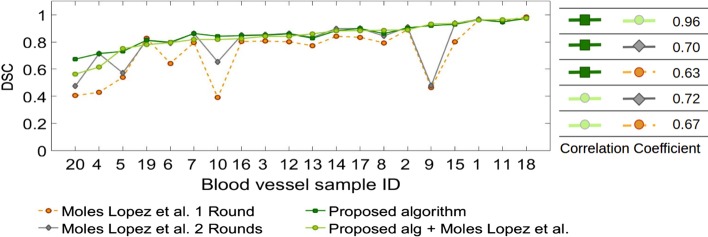
Figure compares registration accuracy using the proposed method, the proposed followed by fine registration (Proposed alg and Moles Lopez et al. [5]), the original (Moles Lopez et al. [5] 1 Round) and the regional version (Moles Lopez et al. [5] 2 Rounds) of the method by Moles Lopez et al. [5] for 5 consecutive slices

**Fig. 5:**
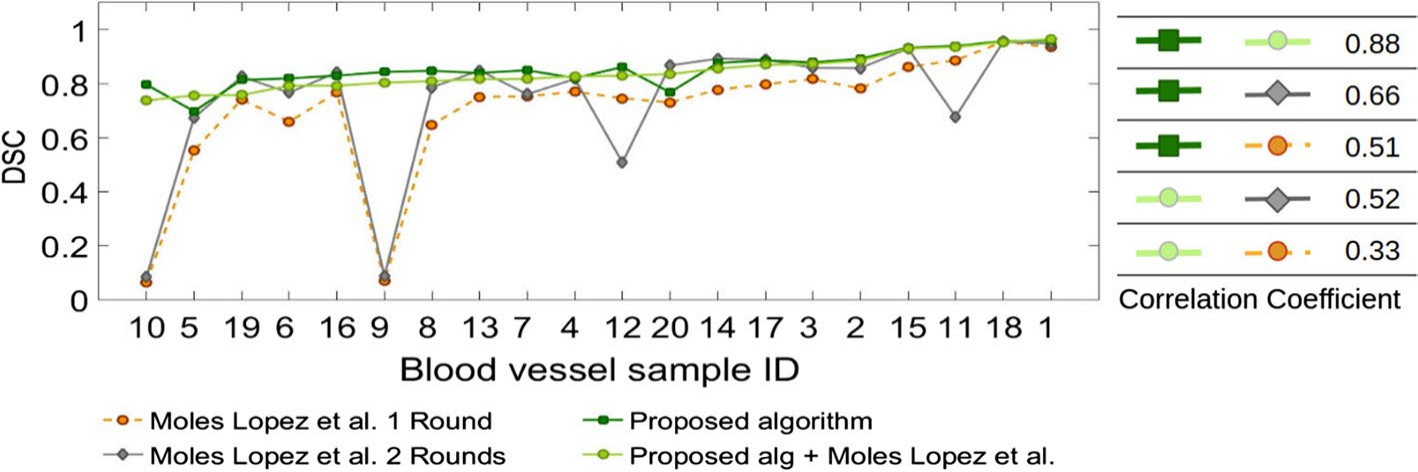
Figure compares the Dice similarity coefficient (DSC) measured for the proposed method and the method proposed by Moles Lopez et al. [5] applied on all slices of the tissue volume (100–150 slices)

Figure 4 compares registration accuracy of the proposed method and the proposed method followed by fine registration using the method by Moles Lopez et al. [5] with those of the whole tissue registration method (Moles Lopez et al. [5] 1 Round) and the regional version of this method (Moles Lopez et al. [5] 2 Rounds) for each blood vessel for 5 consecutive slices. As shown, both the proposed method and the proposed algorithm followed by fine registration provide more accurate and robust results. We also performed registration for all tissue slices (100–150) for the 20 blood vessels using the proposed algorithm and compared the results with the three other methods in Fig. 5.

The measured DSC for the proposed algorithm and the proposed algorithm followed by fine registration was $0.84 \pm 0.07$, and $0.86 \pm 0.06$, respectively, both outperforming the work of Moles Lopez et al. [5] ($0.70 \pm 0.24$) and the regional version of this algorithm ($0.74 \pm 0.25$) for all 100-150 slices. The fourth and fifth columns in Figs. 2 and 3 show 3D reconstruction of the registered lumen masks using the proposed algorithm followed by fine registration, and regional version (Moles Lopez et al. [5] 2 rounds) of the method by Moles Lopez et al. [5].

In order to ensure that the proposed registration algorithm and the final registration results after applying fine registration are robust against different sizes of the ROI, we considered different ROI sizes around the blood vessels of interest and performed registration using the proposed algorithm and the competing methods. The results are provided in Additional file 1: Fig. 4. The results suggest that performing fine registration after applying the proposed algorithm does not always provide reasonable results for small ROIs for the experiments with $MSL = 10$. However, fine registration is robust against medium and large ROIs with $MSL=10$ and all three ROI sizes with $MSL = 1$.

### Comparison with a patch-based deep learning approach

**Fig. 6:**
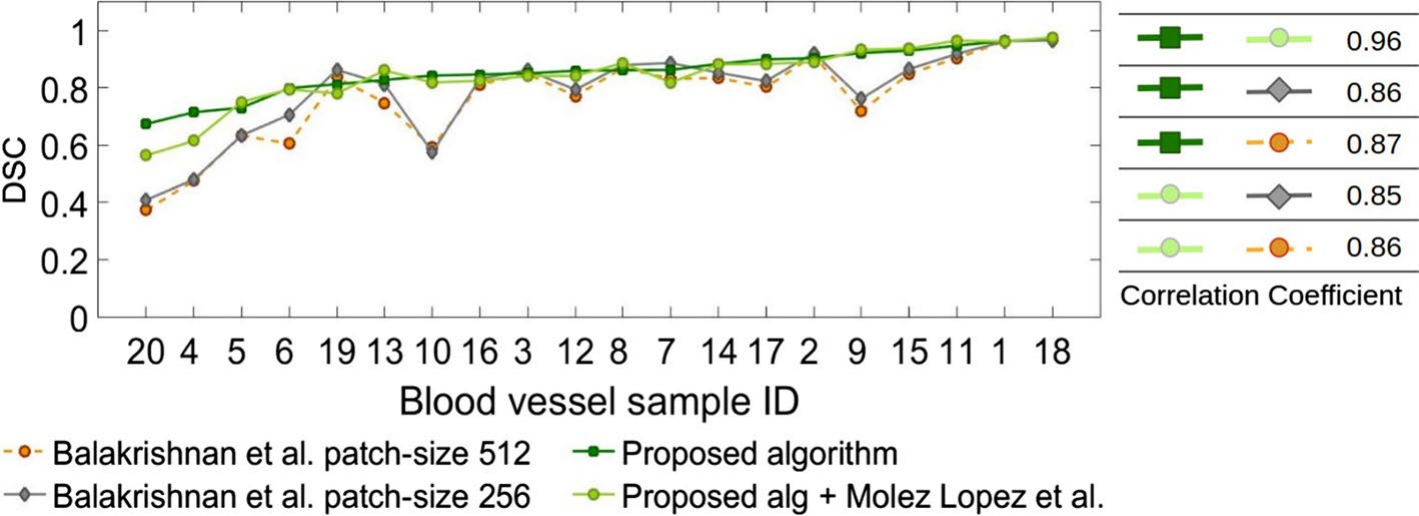
Figure compares the Dice similarity coefficient (DSC) measured for the proposed method and the proposed method followed by fine registration (Proposed alg and Moles Lopez et al. [5]), and the patch-based registration method by Balakrishnan et al. [7] with patch sizes 256 and 512

**Fig. 7:**
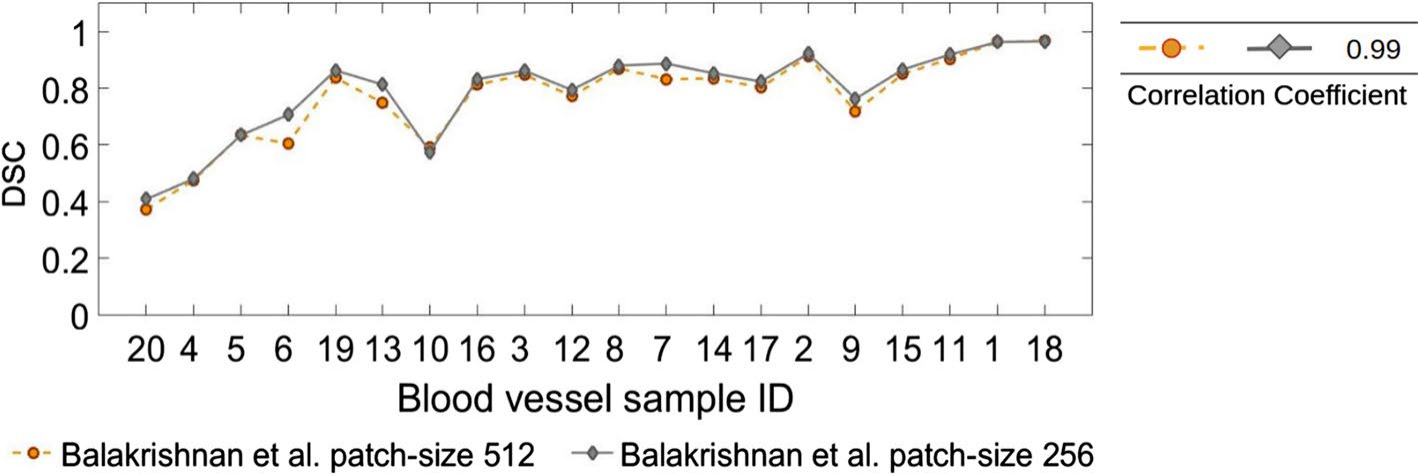
Figure shows the registration results for the patch-based registration method [7] with patch sizes 256 × 256 and 512 × 512

We also compared our algorithm to the patch-based registration algorithm proposed by Balakrishnan et al. [7]. We first registered all slices in an affine manner using the Fiji tool [21]. Later, we trained the proposed neural network model with pairs of patches extracted from a patient’s image scans and applied the trained model on the images from the same patient to have a fair comparison with the other methods. In order to register 5 consecutive image slices for each patient, we first trained the proposed convolutional neural network model in an unsupervised manner on randomly selected patches cropped from these image slices. The trained model was then applied on the same consecutive pairs of whole slide images to register. Hyperparameters were tuned to get high registration accuracies. The values were assigned as follows. Two models were trained with patch-sizes of 256 $\times$ 256 and patch-sizes of 512 $\times$ 512 with a batch-size of 8 and 500 pairs of patches sampled in each iteration. The regularization parameter value for the loss function was set to 1, a learning rate of $1e-3$ was defined and mean squared error was utilized as the image similarity metric. Both models were trained for 30 iterations. The number of iterations was found to be enough for both models to converge and keep the required training time short. The results for both patch-sizes were compared with our proposed registration results in Fig. 6. The registration results for the patch-based registration algorithm by Balakrishnan et al. [7] for different patch-sizes are also shown separately in Fig. 7. As can be seen, the patch-based registration algorithm is not able to provide a good registration of the ROI compared to our proposed method.

### Comparison with a SIFT-feature-based non-rigid approach

**Fig. 8:**
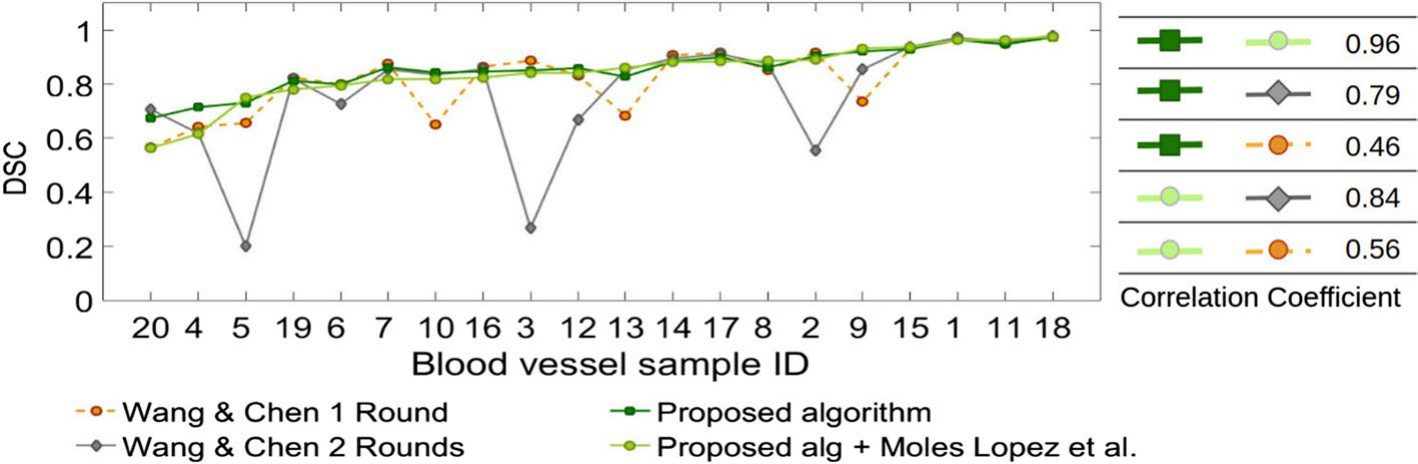
Figure compares our results with the original (Wang and Chen [6] 1 Round) and the regional version (Wang and Chen [6] 2 Rounds) of the proposed method by Wang and Chen [6] for 5 consecutive slices

We also compared the proposed algorithm to whole tissue registration method by Wang and Chen [6] mentioned in Background Section which, similar to our algorithm, uses SIFT features for registration. In order to improve the registration results, they also perform an area-based bi-directional elastic b-spline registration as the final stage of their pipeline. In a similar way, we performed registration for the 20 defined blood vessels for 5 consecutive slices using the proposed method by Wang and Chen [6] (Wang and Chen [6] 1 Round) and the regional version of this method (Wang and Chen [6] 2 Rounds) and compared the results with those of our proposed method in Fig. 8. Quantitative comparison is also provided in Table 2. As can be seen, our algorithm outputs more robust results with better or similar accuracy supporting our assumption that a rigid registration is sufficient for local registration of the tissue.

The fourth column in Table 2 reports the measured execution time for registration of 5 consecutive slices by the corresponding algorithm. Note that the average execution time for the proposed algorithm followed by Moles Lopez et al. [5] is less than the average execution time for the original method [5] because fine registration using [5] is performed on the ROI which takes a shorter time compared to when running the algorithm on the whole image. A more optimal incorporation of the two algorithms together can further decrease the execution time for the proposed algorithm followed by Moles Lopez et al. [5]. Also, note that the measured time for the original and regional version of the method by Wang and Chen [6] was calculated on a Windows 8.1 Pro 64-bit system with Intel Core i7-4720HQ CPU 2.60GHz and 11.9GB RAM while the rest of the experiments were carried out on an Ubuntu 19.04 LTS 64 bit system with Intel Core i7-6700 CPU 3.40 GHz × 8 with 31.4GB RAM for technical reasons. The third column in the Table for the patch-based method by Balakrishnan et al. [7] refers to the average time spent to train the model using randomly extracted patches from the input image slides.

It should also be mentioned that the measured execution time does not include the amount of time required by the user to select the ROI for each blood vessel of interest. However, this task is trivial and usually takes a few seconds for the following reason. Selection of the ROI is performed in a lower resolution of the whole tissue slide such that the user is able to see the blood vessels clearly and at the same time have a complete view of the whole tissue. The selected ROI by the user in this resolution is then upsampled to find the ROI for the full resolution of the image. The size of the ROI in the full resolution is then used for all lower resolutions unless the ROI boundaries exceed the low-resolution image boundaries, in that case the ROI is adjusted to stay in the low-resolution image boundaries.

## Discussion

In this paper, we propose a novel multi-scale approach for registration of the tissue in whole slide images. The user marks the ROI in the tissue section. The proposed algorithm will perform registration for that ROI in consecutive whole slides images. We use a novel multi-scale attention mechanism that concentrates on a big region around the user-defined ROI in low resolutions of the images and incrementally gives more attention to smaller regions around the user-defined ROI in higher resolutions. We also develop a method that effectively selects a subset of SIFT key features that can align the two regions very well. The registration outcome is not affected by artifacts outside of the ROI.

Our experiments approve the fact that simple rigid transformation models can result in better regional registrations even in the presence of non-rigid deformations. In other words, the transformation field for a non-rigidly deformed tissue can be approximated by many small rigid deformations measured on small sections on the tissue.

The proposed method is staining-invariant and can be applied on multi-stained, double-stained, or Luminance images since it takes advantage of coarse features that are extracted using SIFT feature detection algorithm. The ability to perform the registration on full-resolution of images increases accuracy of the results. Our experiments showed that the proposed method outperforms two state-of-the-art rigid and non-rigid registration algorithms and one deep-learning, patch-based registration algorithm.

Although the proposed method was not evaluated on different image modalities in this paper, deploying this method for different modalities is straight-forward as the only parameter that needs to be tuned is the number of layers in each octave for the SIFT feature detection algorithm which effects the number of detected key points on the tissue. For our experiments, we used the same number of layers per octave (10) for all the patient scans.

Despite the importance of registering whole slide images before performing tissue analysis, the work on whole slide image registration is quite limited. In this work, we tried our best to cite the most recent works on whole slide image registration and to compare our algorithm with the state-of-the-art methods.

It should be noted that in the existence of highly deformed regions in the tissue slice, it is not possible to propose a global deformation field for the whole tissue. Existing patch-based registration methods such as the works by Roberts et al. [10] and Liang et al. [9] provide solutions for merging deformation fields acquired for different image patches. However, they do not take into account the existence of highly deformed regions for which the registration results may be considerably poor. Therefore, merging deformation fields of the regions of interests in the existence of highly deformed regions in the tissue slice needs to be addressed. Future work will address this problem. To make the algorithm more deployable, an automatic or semi-automatic method for detection of the ROIs would be desirable. Finally, measures should be taken to speed up the proposed method for near real-time applications.

## Conclusion

In order to study the tumor tissue in terms of vasculature and cell population, 3D reconstruction of the sliced and imaged tumor volume is necessary. We proposed a multi-scale registration algorithm which provides more robust and accurate results compared to two linear and nonlinear whole slide image registration algorithms and one patch-based registration algorithm. The better registration accuracy of our proposed method can be attributed to the novel multi-scale attention mechanism that was deployed which incrementally focuses registration on a smaller region around the region of interest in higher resolutions. Moreover, the proposed method needs only minor parameter tuning. Future work includes analysis of the reconstructed tissue volumes by the proposed algorithm to study drug influence on angiogenesis and cell populations in tumors. The next section provides details on different steps of the proposed algorithm.

## Method

**Fig. 9:**
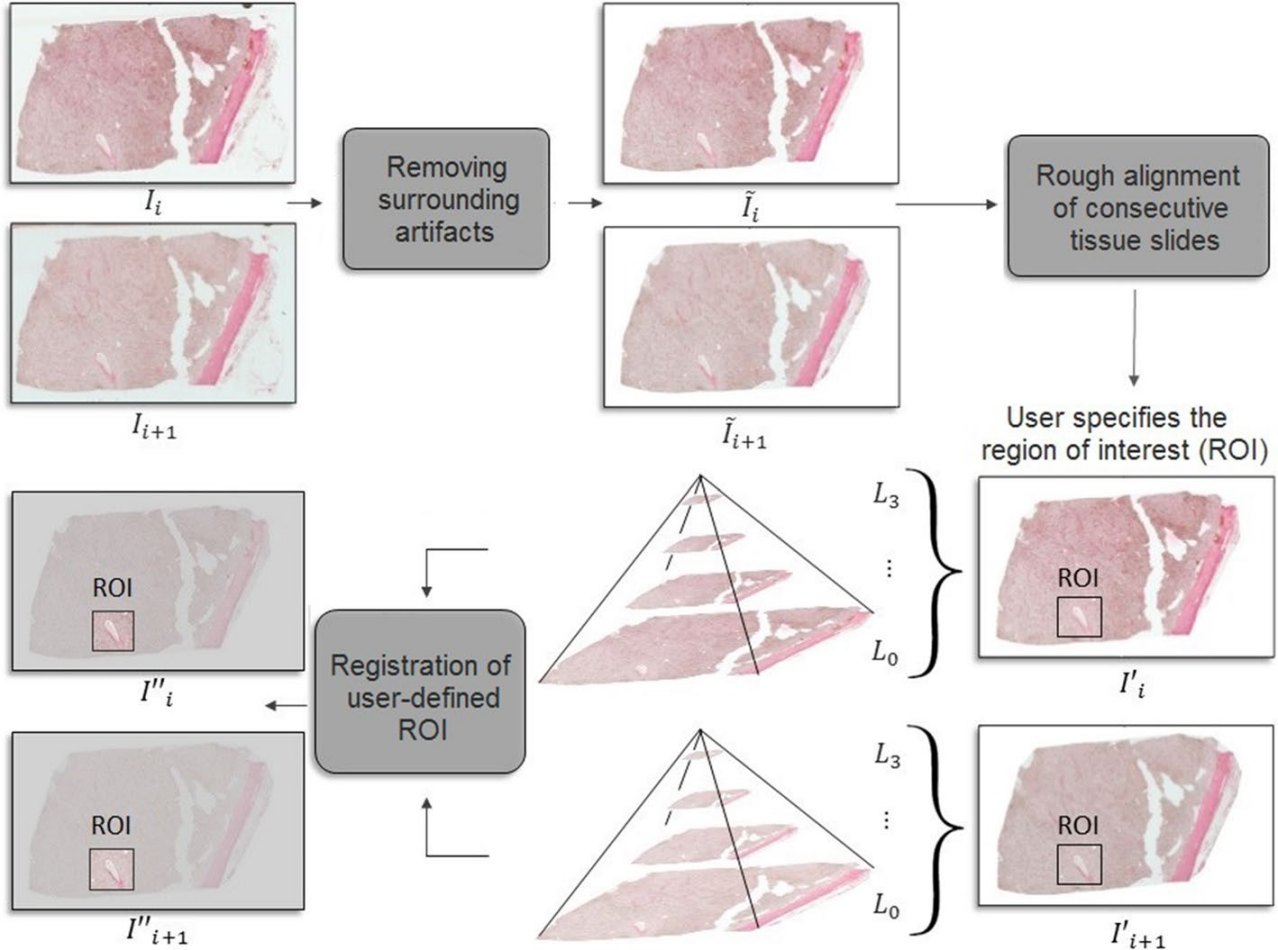
The diagram shows an overview of the proposed algorithm for regional registration of whole slide images. The *Removing surrounding artifacts* step removes the extra stains and artifacts around the tissue. The *Rough alignment of consecutive tissue slides* step roughly aligns the whole tissue in consecutive whole slide images. Finally, the ROI marked by the user is registered in consecutive slides using a multi-scale approach in the *Registration of the user-defined ROI* step

In order to register a user-defined ROI in the whole slide image stack, three steps are carried out as follows: (1) removing surrounding artifacts, to remove extra stains and artifacts around the tissue, (2) rough alignment of consecutive tissue slides, to approximately align the whole tissue in consecutive whole slide images, (3) registration of the user-defined ROI, to register the ROI marked by the user. Finally, fine registration is carried out to improve the registration for the ROI. Figure 9 gives an overview of the steps of the proposed method. A flowchart for each step is provided in Additional file 1: Figs. 1 and 2.

### Removing surrounding artifacts

This step of the registration algorithm is done on a single scale. Extra stains and artifacts around the tissue can affect the registration outcome. To remove these artifacts, each image ($I_i$) is converted to the gray scale and smoothed using a Gaussian filter with a standard deviation of 10 pixels. The smoothed image is then thresholded using a threshold value equal to the mean pixel intensity of the image. Since an accurate segmentation of the tissue from the surrounding artifacts cannot be achieved merely by thresholding, an opening and later a closing morphological operation was applied on the output mask from thresholding using a circular kernel of radius 20 pixels to get a mask that covers the artifacts and extra stains around the tissue. The final segmentation mask is then applied to the image to remove the surrounding artifacts. Contours in the new image are then detected. The contours which are closer to the center of the image and surround the largest area in the image are identified. Extra tissue and stains outside the convex hull of the selected contours are removed, resulting in a cleaned tissue image ($\tilde{I}_i$). In the next step, rough registration of the whole tissue is carried out for consecutive whole slide images.

### Rough alignment of consecutive tissue slides

This step of the registration algorithm is also done on a single scale. In this stage, consecutive whole tissue slides are roughly aligned by correcting relative rotations or displacements in the location of the tissue across consecutive virtual slides. The cleaned image, $\tilde{I}_i$, is segmented using the multi-resolution Monte Carlo method of Sashida et al. [22] which performs piece-wise constant segmentation of the Mumford and Shah [23] model to yield $\tilde{I}_i^{MS}$. Mumford-Shah segmentation of the image removes the noise, texture and small spatial intensity variations making the image clean and the upcoming registration robust against inter-slice intensity variations due to differences in stain densities. We chose Sashida’s approach for segmentation of the Mumford-Shah model mainly due to its outperformance over other approaches such as the work by Song and Chan [24] and Bae and Tai [25] in multi-phase segmentation of images. Next, each consecutive pair of Mumford-Shah segmented images are registered independently. For each pair of images $\{\tilde{I}_i^{MS},\tilde{I}_{i+1}^{MS}\}$, a combination of varying translation (*dx*, *dy*) and rotation ($\theta$) transformations are applied to the second (moving) image to find the rotation and translation parameters which make $T_{\theta ,dx,dy}[\tilde{I}_{i+1}^{MS}(x,y)]$ most similar to $\tilde{I}_i^{MS}(x,y)$ by optimizing the following function:

1 $$\begin{aligned} \mathop {\mathrm{arg min}}_{\{\theta ,dx,dy\}}\sum _{x,y} \left( T_{\theta ,dx,dy}[\tilde{I}_{i+1}^{MS}(x,y)]-\tilde{I}_{i}^{MS}(x,y)\right) ^2 \end{aligned}$$

The $\{\theta , dx, dy\}$ triplet which gives the least sum of squared difference is chosen and its corresponding transformation matrix is applied to the moving image: ${I'}_{i+1} \leftarrow T_{\theta ,dx,dy}[\tilde{I}_{i+1}]$. These two steps roughly align the images in consecutive image slides. Other algorithms which can be used for rough registration of the whole tissue in subsequent image slices include landmark-based registration methods or b-spline registration algorithms. For the same reason mentioned in the Background Section, landmark-based methods may not provide a fairly accurate initial registration in case the selected landmark is located in a tissue section which experiences severe deformations in certain image slices in the WSI stack. Moreover, optimization of the parameters for a b-spline transformation technique is computationally demanding. A number of parameters specify the appearance and complexity of B-spline curves such as the number of control points and their relative location. These values are usually tuned using trial-and-error procedures. Many algorithms have been proposed for parameter optimization for B-spline curve fitting [26, 27]. Parameter optimization gets especially more challenging for the case of discontinuous control points [28] which can occur in the existence of major distortions. Therefore, a linear transformation technique was utilized in this step. In the next step, the ROI is registered in consecutive image slices.

### Registration of user-defined ROI

Multi-scale registration is utilized at this stage of the registration algorithm. Registration of the whole tissue provides a fairly accurate initial alignment for further registration of the ROI. To decrease the adverse influence of tissue deformations outside of the ROI, a multi-scale registration approach that incrementally confines its attention to a smaller region around the ROI is deployed at this stage. Different steps of this approach are explained below:

#### Multi-scale attention

**Fig. 10:**
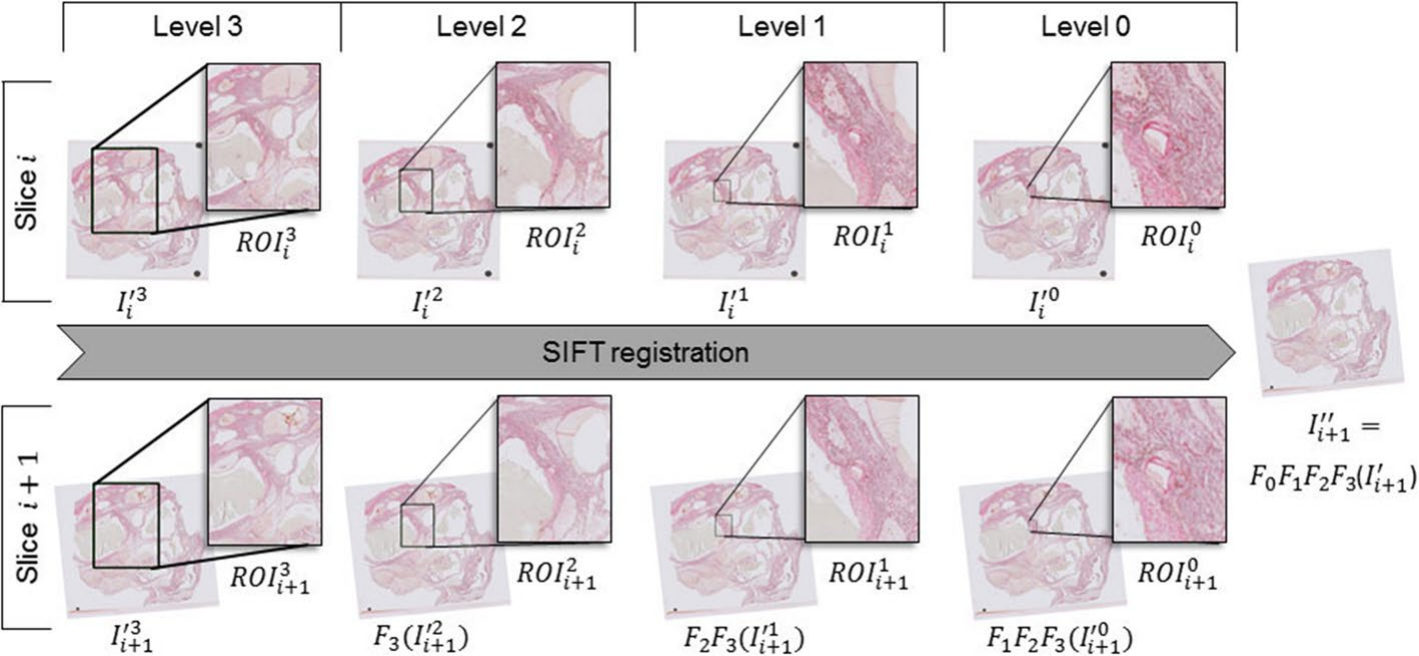
Figure shows the ROI in multiple resolutions (levels 0 to 3 with level 0 referring to the highest resolution of the image slices) of two consecutive whole slide images. The user defines the $ROI^0_i$ for the target image in its highest resolution ($I^0_i$). The ROI in lower resolutions ($ROI^{1,2,3}_i$) are defined automatically. $F_r$ refers to the best rigid transformation matrix found to align slice $i+1$ to slice *i* in resolution level *r* of the slices

Let us define the registered image sequence from the previous step as ${I'}_{seq} = \{{I'}_1, {I'}_2, \ldots \}$. A small box around the ROI is defined by the user for the image at its full resolution ($r=0$) as $ROI^{r=0} \equiv ROI^{0}$ (see $ROI_i^{0}$ in Fig 8). In order to register $ROI^{0}$ in ${I'}_i$ and ${I'}_{i+1}$ image slices, registration is first performed for lower resolutions of the two images. ${I'}_i^{r=k}$ ($\equiv {I'}_i^{k}$) is denoted as the output image from downsampling ${I'}_i$ by a factor of $2^k$. Similarly, a downsampled image is generated for image ${I'}_{i+1}$ and is denoted as ${I'}_{i+1}^{k}$. Registration is performed between ${I'}_{i}^{k}$ and ${I'}_{i+1}^{k}$ by considering $ROI^{k}$ only. The same procedure is taken for $r = \{k-1, k-2, \ldots , 0\}$ in the presented order giving a series of rigid transformations ($F_{r}$) as follows:

2 $$\begin{aligned} SIFT_{{ROI}^k}\left( {I'}_{i}^{k}, {I'}_{i+1}^{k}\right)\rightarrow & {} F_{k} \nonumber \\ SIFT_{{ROI}^{k-1}}\left( {I'}_{i}^{k-1}, F_{k}\left( {I'}_{i+1}^{k-1}\right) \right)\rightarrow & {} F_{k-1}F_{k} \nonumber \\ \vdots \nonumber \\ SIFT_{{ROI}^{0}} \left( {I'}_{i}^{0}, F_{1}\dots F_{k}\left( {I'}_{i+1}^{0}\right) \right)\rightarrow & {} F_{0} \ldots F_{k-1} F_{k} \end{aligned}$$

where $SIFT_{ROI} (I,J)$ performs registration for the specified *ROI* in the image pair (*I*, *J*) using SIFT features and will be explained shortly. The resulting transformations are applied to image ${I'}^0_{i+1}$. The ROI for different image resolutions is defined as follows: If $(x_{ROI},y_{ROI})$ is the center coordinates of $ROI^{0}$ in ${I'}_i^{0}$, the corresponding center coordinates in the downsampled image ${I'}_i^{k}$ are calculated as $(x_{ROI}/2^k,y_{ROI}/2^k)$. The ROI ($ROI^{k}$) for ${I'}_i^{k}$ is then extracted with the same width and height as for $ROI^{0}$ unless the ROI boundaries exceed the image boundaries. In that case, the width and height are adjusted such that the ROI stays in the image boundaries. Figure 10 shows the extracted ROI from different image resolutions for the manually identified ROI ($ROI^0_i$) in the full resolution image (${I'}^0_i$). The novel difference between the proposed method and the existing multi-scale registration methods lies in this step, the proposed algorithm confines its attention to a large region around the original user-defined ROI ($ROI^{0}$) in the low-resolution images and focuses its attention to a smaller region around the user-defined ROI as the resolution of the images increase. The multi-scale nature of the algorithm makes it computationally efficient. Next section describes the *SIFT* function in Eq. 2 which registers the *ROIs* in each resolution.

#### Registration using SIFT features

Having extracted the ROI $ROI^k$ in the low-resolution images ${I'}^{k}_i$ and ${I'}^{k}_{i+1}$, distinctive key points are detected in both ROIs using SIFT feature detection algorithm [14]. Spatial coordinates of the detected key points are added to the descriptors of the key points and are taken into account for key point matching in the two ROIs. From all the identified pairs of SIFT key matches, 8 strong matches are found for the two ROIs. If the number of identified matches are less than 8, all the SIFT key matches are selected. Since registration is performed locally, a rigid registration is found sufficient. A rigid transformation can be calculated with a minimum of 3 key points per image. Therefore, $$\left( {\begin{array}{c}8\\ 3\end{array}}\right) =56$$ different combinations of 3 matches and consequently, 56 different transformation matrices can be obtained using the 8 selected matches. Our experiments show that these combinations provide a sufficiently diverse set of transformations from which an accurate registration can often be achieved. All the transformations are applied to the previously registered image ${I'}_{i+1}^{k}$ in the *Rough alignment of consecutive tissue slides* Section giving a series of 56 warped images

3 $$\begin{aligned} \left\{F_1\left( {I'}_{i+1}^{k}\right) , F_2\left( {I'}_{i+1}^{k}\right) , \ldots , F_{56}\left( {I'}_{i+1}^{k}\right) \right\} \end{aligned}$$

where $F_m$ is the transformation matrix found by using a unique combination of 3 matches. From the 56 transformation proposals, the transformation matrix which gives the least sum of squared difference in pixel intensity (*D*) for $ROI_k$ in the warped image $F_m\left( {I'}_{i+1}^{k}\right)$ and the reference image ${I'}_{i}^{k}$ is chosen using the following function:

4 $$\begin{aligned} F_k = \displaystyle \min _{F_m} D_{ROI_k}\left( {I'}_{i}^{k}, F_m({I'}_{i+1}^{k})\right) , \forall m \in [1, 56] \end{aligned}$$

The best transformation matrix found for resolution *k* is then scaled up and applied to the moving image in the higher resolution ${I'}_{i+1}^{k-1}$. This process is repeated for resolutions $r = \{k-1, k-2, \ldots , 0\}$ in the presented order and the registration for the image pair (${I'}_{i}$, ${I'}_{i+1}$) is finalized by defining the transformation matrix for $r=0$ as $F^* = F_0 F_1 \ldots F_k$ and applying it to the original moving image: ${I''}_{i+1} \leftarrow F^*({I'}_{i+1})$. Similarly, registration is performed for the registered image ${I''}_{i+1}$ and the next image in the stack (${I''}_{i+1}$, ${I'}_{i+2}$) aligning the ROI in the entire image stack: ${I''}_{seq} = \{{I''}_1, {I''}_2, \ldots , {I''}_{i+1}, {I''}_{i+2}, \ldots \}$. A diagram of this process is shown in Fig. 10. Note that as the algorithm steps through higher resolutions of the images, the SIFT key points are extracted from a smaller region around the user-defined ROI. Hence, the proposed algorithm stays robust even in the existence of distorted tissue regions that are outside of the ROI marked by the user.

## Supplementary information

**Additional file 1**: Provides detailed flow charts of different stages of the registration algorithm: (1) removing surrounding artifacts, (2) rough alignment of consecutive tissue slides, and (3) registration of the ROI. Also, examples of the best SIFT feature matches for the ROIs in the previous and current slides, and the selected 3 SIFT feature matches that resulted in the best alignment of the 2 slides are also provided. Finally, the experimental results are presented supporting that different ROI sizes around the blood vessels of interest output reasonable registration results.

## Data Availability

The proposed algorithm is available for download at this site: https://github.com/MahsaPaknezhad/WSIRegistration. The data that support the findings of this study are available from Singapore General Hospital but restrictions apply to the availability of these data, which were used under license for the current study, and so are not publicly available. A supplementary document (SupplementaryMaterials.pdf) accompanies this manuscript.

## Abbreviations

WSI: Whole slide images
IHC: Immunohistochemistry
ROI: Region of interest
SGD: Stochastic gradient descent
MI: Mutual information
NCC: Normalized cross correlation
MSL: Maximum step length
