# Supplementary material for: Regional registration of whole slide image stacks containing major histological artifacts
Source: BMC Bioinformatics. 2020 Dec 4;21:558. doi: 10.1186/s12859-020-03907-6 (PMC7718714; doi:10.1186/s12859-020-03907-6)
Supplement: Supplementary file 1 — Additional file 1: Provides detailed flow charts of different stages of the registration algorithm: (1) removing surrounding artifacts, (2) rough alignment of consecutive tissue slides, and (3) registration of the ROI. Also, examples of the best SIFT feature matches for the ROIs in the previous and current slides, and the selected 3 SIFT feature matches that resulted in the best alignment of the 2 slides are also provided. Finally, the experimental results are presented supporting that different ROI sizes around the blood vessels of interest output reasonable registration results. [file 12859_2020_3907_MOESM1_ESM.pdf]

Supplementary Materials

October 20, 2020

Fig. (1) shows the flowcharts of the *Removing surrounding artifacts* and *Rough alignment of consecutive tissue slides* steps of the proposed algorithm. Fig. (2) shows the flowchart for the *Registration of user-defined ROI* of the algorithm.

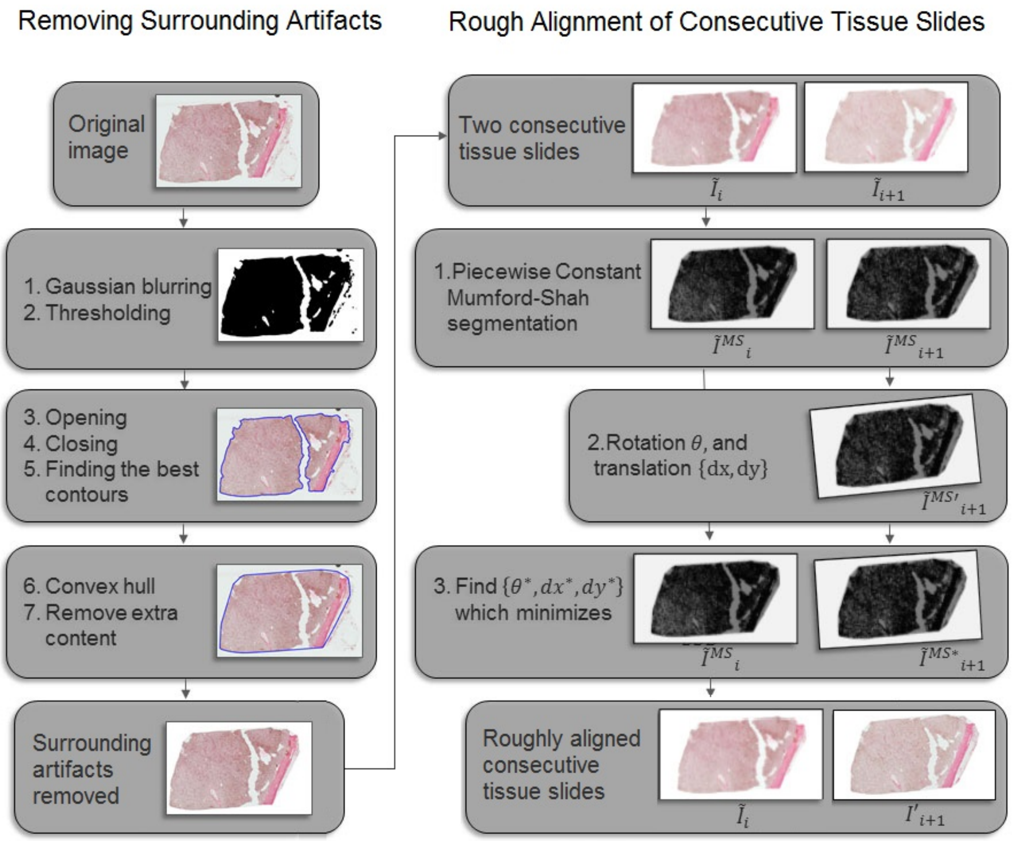

Figure 1: Figure shows in more details the steps that are carried out in the *Removing surrounding artifacts* and *Rough alignment of consecutive tissue slides* stages of the proposed algorithm.

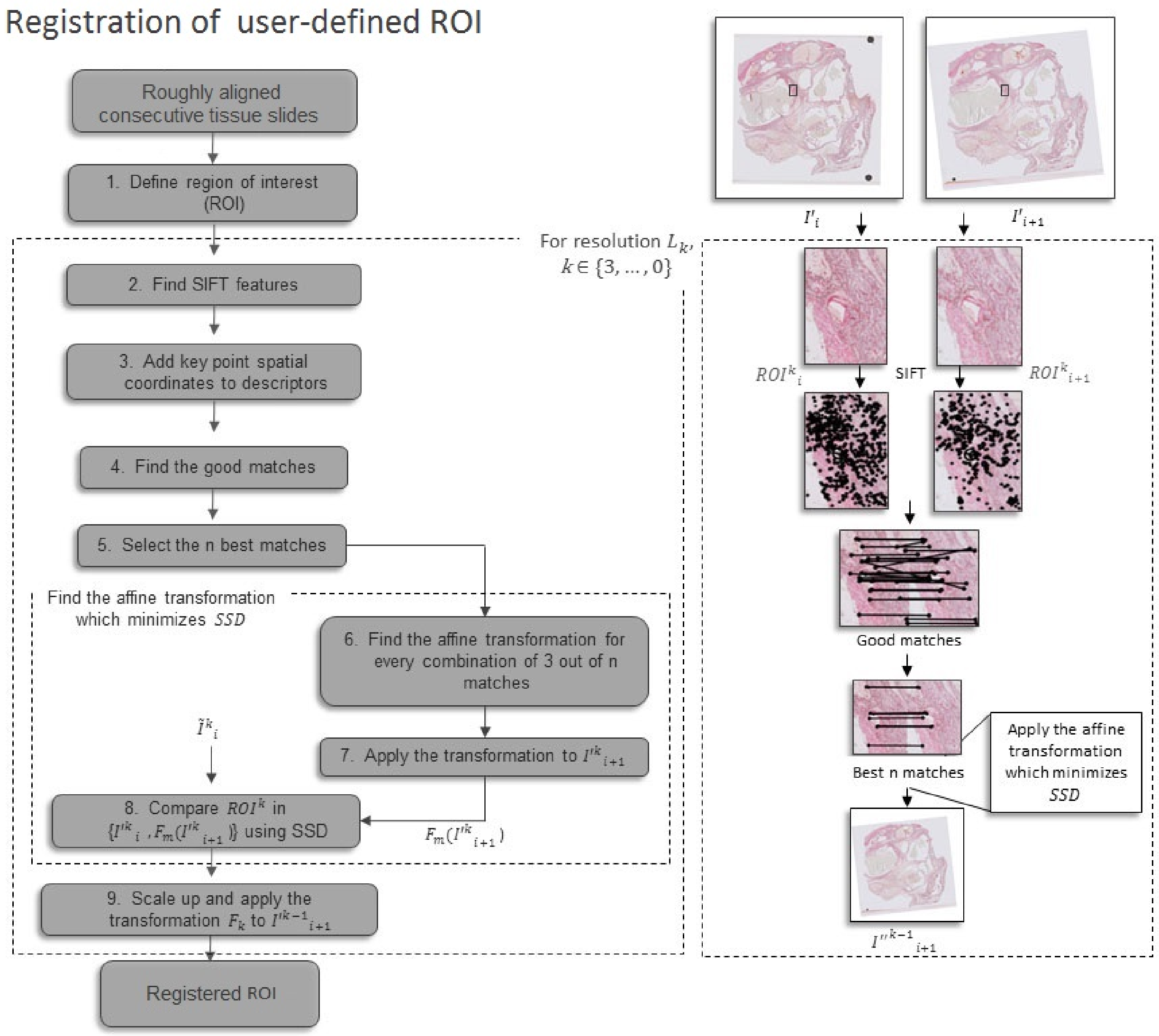

Figure 2: Figure shows the steps that are taken in the *Registration of user-defined ROI* stage of the algorithm to register the ROI for subsequent whole slide images.

Fig. 3 shows the SIFT registration results for a few pairs of consecutive slides for different blood vessels. For each example, the top left image shows the best SIFT feature matches for the ROIs in the two consecutive slides. Also shown is the selected 3 SIFT matches that resulted in the best alignment of the 2 slides. The image on the right shows the segmentation for the moving slide (blue) mapped on the fixed slide after performing registration. The segmentation for the fixed slide is also shown in green. One can see that the two slides are aligned fairly well.

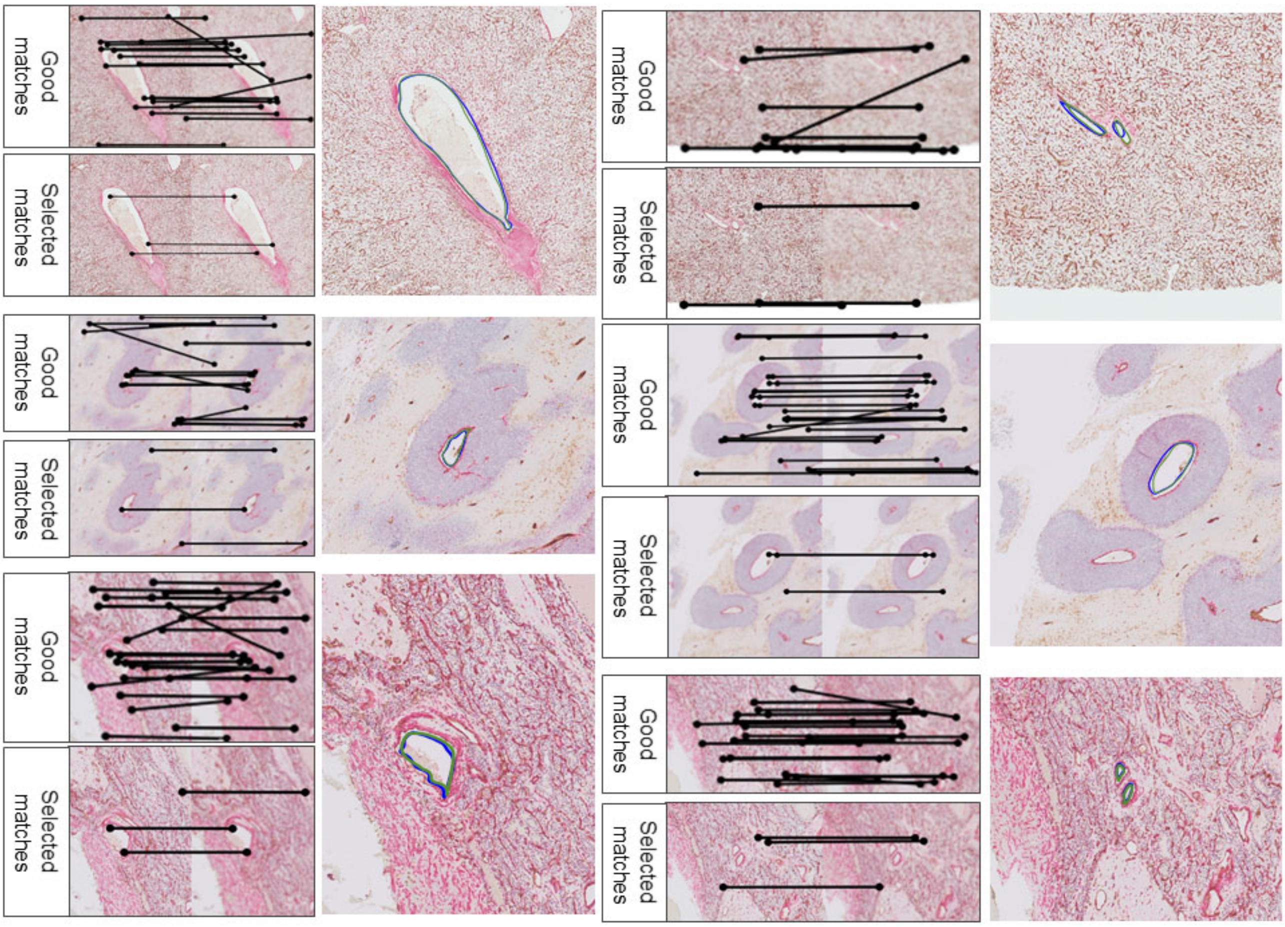

Figure 3: Figure shows a few examples of the SIFT registration results. For each example, the left top figure shows the matched SIFT features for the full resolution ROI in the two consecutive slides ($ROI^0_{i,i+1}$). The bottom left figure shows the three SIFT matches that led to the best alignment between the two slides. The right figure shows segmentation for the moving slide (blue) mapped on the fixed slide after performing registration. Also shown is the segmentation for the fixed slide (green). One can see that the two segmentations are reasonably aligned.

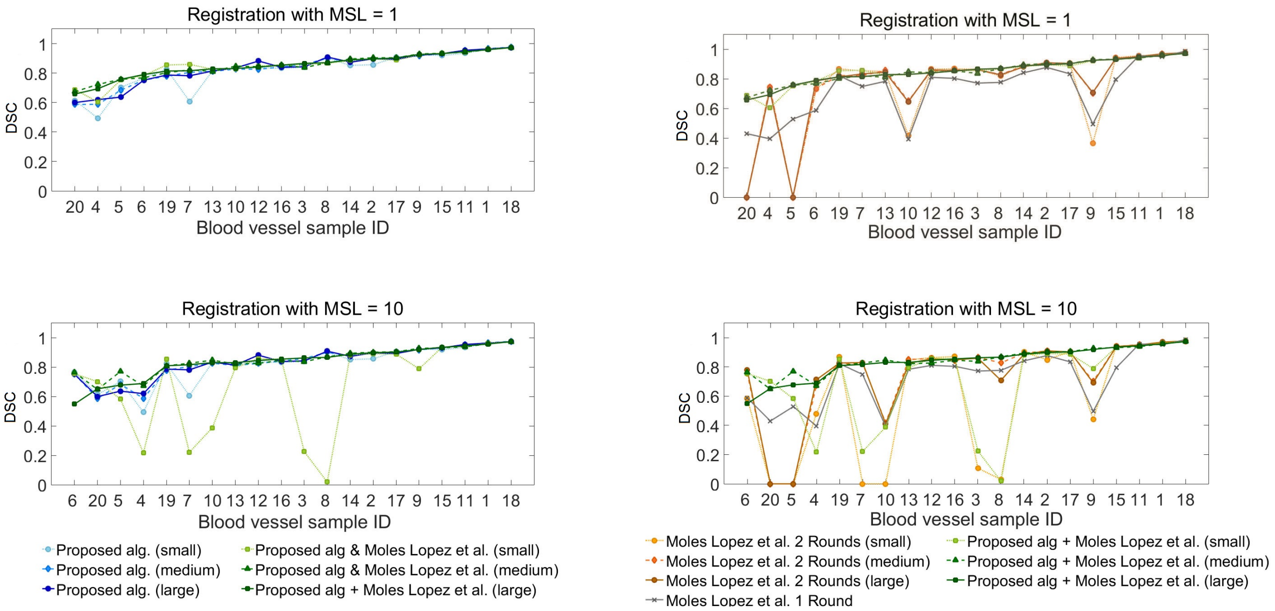

Figure 4: (Left) shows the registration accuracy using the proposed registration algorithm using different ROI sizes. (small) and (large) refer to the smallest and largest ROIs considered for the blood vessels, respectively (with (large) ROI being 150 pixels bigger than the (small) ROI in both dimensions). Proposed alg. + [2] shows the registration accuracy for the proposed registration algorithm followed by fine registration using different ROI sizes. (Right) compares the registration accuracy with those of the competing algorithms ([2] 1 Round and 2 Rounds). (Top row) shows the results using $MSL = 1$ for fine registration. (Bottom row) shows these results for fine registration using $MSL = 10$.

To ensure that the proposed registration algorithm and the final registration results after applying fine registration are robust against different sizes of the region of interest (ROI), we considered three different ROI sizes around the blood vessels of interest and performed registration using the proposed algorithm and the competing methods. After applying the proposed algorithm, fine registration was carried out using the work of [2] with the best identified parameter values: $T_\mu = Affine$, $S = MI$, $MSL = 10$, and $Input = Lum$ and another set of values with smaller MSL: $T_\mu = Affine$, $S = MI$, $MSL = 1$, and $Input = Lum$. As shown in Fig. 4 (Left), the results suggest that performing fine registration after applying the proposed algorithm does not always provide reasonable results for the small ROIs for the experiments with $MSL = 10$. However, fine registration is robust against medium and large ROIs with $MLS = 10$ and all three ROI sizes with $MSL = 1$. Fig. 4 (Right) compares the registration accuracy for the proposed registration method followed by fine registration with those of the whole tissue registration method ([2] 1 Round) and the regional version of this method ([2] 2 Rounds) using $MSL = 1$ and $MSL = 10$ for fine registration. One can see that the registration outputs are more accurate and robust for larger ROI sizes and the algorithm performs better using $MSL = 1$.

References

[1] G Balakrishnan, A Zhao, M R Sabuncu, J Guttag, and A V Dalca. Voxelmorph: a learning framework for deformable medical image registration. *IEEE transactions on medical imaging*, 38(8):1788–1800, 2019.

[2] X Moles Lopez, P Barbot, Y Van Eycke, L Verset, A L Trépant, L Larbanoix, I Salmon, and C Decaestecker. Registration of whole immunohistochemical slide images: an efficient way to

characterize biomarker colocalization. *Journal of the American Medical Informatics Association*, 22(1):86–99, 2014.

[3] C W Wang and H C Chen. Improved image alignment method in application to x-ray images and biological images. *Bioinformatics*, 29(15):1879–1887, 2013.
